# Detection of IgM and IgG antibodies in patients with coronavirus disease 2019

**DOI:** 10.1002/cti2.1136

**Published:** 2020-05-06

**Authors:** Hongyan Hou, Ting Wang, Bo Zhang, Ying Luo, Lie Mao, Feng Wang, Shiji Wu, Ziyong Sun

**Affiliations:** ^1^ Department of Laboratory Medicine Tongji Hospital Tongji Medical College Huazhong University of Science and Technology Wuhan China

**Keywords:** COVID‐19, illness severity, immunoglobulin G, immunoglobulin M, SARS‐CoV‐2

## Abstract

**Objectives:**

This study aimed to determine the IgM and IgG responses against severe acute respiratory syndrome coronavirus (SARS‐CoV)‐2 in coronavirus disease 2019 (COVID‐19) patients with varying illness severities.

**Methods:**

IgM and IgG antibody levels were assessed via chemiluminescence immunoassay in 338 COVID‐19 patients.

**Results:**

IgM levels increased during the first week after SARS‐CoV‐2 infection, peaked 2 weeks and then reduced to near‐background levels in most patients. IgG was detectable after 1 week and was maintained at a high level for a long period. The positive rates of IgM and/or IgG antibody detections were not significantly different among the mild, severe and critical disease groups. Severe and critical cases had higher IgM levels than mild cases, whereas the IgG level in critical cases was lower than those in both mild and severe cases. This might be because of the high disease activity and/or a compromised immune response in critical cases. The IgM antibody levels were slightly higher in deceased patients than recovered patients, but IgG levels in these groups did not significantly differ. A longitudinal detection of antibodies revealed that IgM levels decreased rapidly in recovered patients, whereas in deceased cases, either IgM levels remained high or both IgM and IgG were undetectable during the disease course.

**Conclusion:**

Quantitative detection of IgM and IgG antibodies against SARS‐CoV‐2 quantitatively has potential significance for evaluating the severity and prognosis of COVID‐19.

## Graphical abstract

In this study, we found that level of IgM was increased during the first week after SARS‐CoV‐2 infection and reached its peak level after 2 weeks, while IgG reached its peak in 3 weeks, which was maintained at a high level even over 48 days.

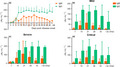

## Introduction

The novel coronavirus, severe acute respiratory syndrome coronavirus (SARS‐CoV)‐2, has been identified as the causative pathogen of coronavirus disease 2019 (COVID‐19).^1^, ^2^, ^3^, ^4^ This disease has been called a public health emergency of international concern by the World Health Organization (WHO). Since December 2019, a serious outbreak of the disease has spread via human‐to‐human transmission from China to more than 200 countries and territories worldwide.^5^, ^6^ The numbers of infected cases and deaths associated with COVID‐19 are still increasing daily. As of 6 April 2020, SARS‐CoV‐2 has caused 1 210 956 confirmed cases and 67 594 deaths worldwide according to the WHO.^6^

The diagnosis of COVID‐19 is dependent mainly on clinical characteristics, CT imaging and a few laboratory tests. Although some symptoms and laboratory parameters have indicative values in confirmed patients, they are not unique to SARS‐CoV‐2 infection. Before the publication of the seventh edition of the ‘Guideline of diagnosis and treatment for COVID‐19’ by the Chinese National Health Commission, laboratory diagnosis of confirmed patients was carried out by detecting viral RNA in throat swab or nasal swab specimens using real‐time reverse transcription polymerase chain reaction (RT‐PCR) assays.^7^ This method does not require live virus to be present in the specimens, but the turnaround times of the current real‐time RT‐PCR assays are long, and these assays need to be performed in certified laboratories. A high percentage of false‐negative results were reported because of the quality of sample collection and multiple preparation steps, limiting the role of this assay for outbreak containment.^8^, ^9^, ^10^, ^11^ Therefore, accurate, convenient and rapid methods are acutely needed for the diagnosis of COVID‐19.

SARS‐CoV‐2 shares similar clinical genetic and epidemiological features with SARS and Middle East respiratory syndrome (MERS).^12^, ^13^ Thus, the process of generating antibodies against SARS‐CoV‐2 might be similar, and the detection of both IgM and IgG antibodies could provide information on the time course of virus infection.^10^, ^14^ Following a SARS infection, IgM is detectable after 3–6 days, and IgG is detectable after 8 days.^15^ Most recently, serological tests for virus‐specific IgM and IgG antibodies against SARS‐CoV‐2 have been developed, and similar serological responses were observed in one COVID‐19 patient.^11^, ^16^ Rapid and specific antibody detection could offer information for confirmation or exclusion of SARS‐CoV‐2 infection in suspected patients and has been recommended by the newest ‘Guideline of diagnosis and treatment for COVID‐19’ issued by the Chinese National Health Commission.^17^

Most COVID‐19 patients have a mild illness and recover quickly after appropriate clinical intervention. Some COVID‐19 patients develop severe SARS, multiple organ failure and even death over a short period of time.^5^, ^18^, ^19^, ^20^ Previous studies have reported that massive inflammatory responses induce the overactivity of T cells, and leads to severe immune injury during SARS‐CoV‐2 infection.^5^, ^18^, ^21^ However, the humoural immune response to COVID‐19 is still largely unknown.

Here, we investigated the production of IgM and IgG detected by a chemiluminescence immunoassay (CLIA) in COVID‐19 patients over the course of their disease.

## Results

The performance of anti‐SARS‐CoV‐2 CLIA‐YHLO kit was verified before its application in our laboratory. Our previous data show that high sensitivity and specificity were observed for this method, and reproductive analysis showed that the coefficient of variation was below 10% (Supplementary figure 1). The present study included a total of 338 hospitalised patients with confirmed COVID‐19; among them, 171 (50.6%) patients were males and 167 (49.4%) were females. The patients were classified into three groups: mild (64 cases, 18.9%), severe (199 cases, 58.9%) and critical (75 cases, 22.2%). The patient ages in the severe (62.79 ± 14.03 years) and critical (66.52 ± 15.6 years) groups were significantly higher than the mild group (55.06 ± 17.78 years). The percentage of males was higher than that of females in the severe and critical groups. Most of the patients had fever, cough, fatigue, expectoration and shortness of breath at illness onset. The most common comorbidities were hypertension (41.1%), diabetes (18.6%), cardiovascular diseases (5.3%) and malignancies (5%) (Table 1). We observed that the critical group had higher percentages of symptom manifestations and comorbidities. By March 10, 232 (68.6%) of the study patients had been discharged, 32 (9.5%) patients had deceased, and 74 (21.9%) were still stay in the hospital. The percentages of recovered patients were higher in the mild and severe groups than in the critical group, and all the deceased cases were in the critical group.

**Table 1:** Baseline characteristics of 338 patients with COVID‐19

	Total (*n* = 338)	Mild (*n* = 64)	Severe (*n* = 199)	Critical (*n* = 75)
Age (years)				
Mean (SD)	62.15 (15.56)	55.06 (17.78)	62.79 (14.03)	66.52 (15.6)
Sex				
Male	171 (50.6%)	27 (42.2%)	102 (51.3%)	42 (56%)
Female	167 (49.4%)	37 (57.8%)	97 (48.7%)	33 (44%)
Signs and symptoms at admission				
Fever	263 (77.8%)	51 (79.7%)	152 (76.4%)	60 (80%)
Cough	152 (45%)	32 (50%)	88 (44.2%)	32 (42.7%)
Fatigue	89 (26.3%)	15 (23.4%)	48 (24.1%)	26 (34.7%)
Expectoration	64 (18.9%)	10 (15.6%)	35 (17.5%)	19 (25.3%)
Shortness of breath	46 (13.6%)	6 (9.3%)	25 (12.5%)	15 (20%)
Chest distress	33 (9.8%)	8 (12.5%)	15 (7.5%)	10 (13.3%)
Diarrhoea	20 (5.9%)	5 (7.8%)	13 (6.5%)	2 (2.7%)
Headache	14 (4.1%)	3 (4.7%)	7 (3.5%)	4 (5.3%)
Nausea and vomiting	11 (3.3%)	3 (4.7%)	6 (3%)	2 (2.7%)
Muscle ache	8 (2.4%)	2 (3.1%)	5 (2.5%)	1 (1.3%)
Pharyngalgia	3 (0.9%)	2 (3.1%)	1 (0.5%)	0
Comorbidities				
Hypertension	140 (41.4%)	20 (31.3%)	85 (42.7%)	35 (46.7%)
Diabetes	63 (18.6%)	6 (9.4%)	42 (21.1%)	15 (20%)
Cardiovascular disease	18 (5.3%)	4 (6.3%)	9 (4.5%)	5 (6.7%)
Malignancy	17 (5%)	2 (3.1%)	11 (5.5%)	4 (5.3%)
COPD	12 (3.6%)	1 (1.6%)	7 (3.5%)	4 (5.3%)
Cerebrovascular disease	11 (3.3%)	2 (3.1%)	6 (3%)	3 (4%)
Tuberculosis	7 (2.1%)	1 (1.6%)	3 (1.5%)	3 (4%)
Digestive system disease	7 (2.1%)	2 (3.1%)	3 (1.5%)	2 (2.7%)
Chronic liver disease	5 (1.5%)	1 (1.6%)	2 (1%)	2 (2.7%)
Chronic kidney disease	3 (0.9%)	0	1 (0.5%)	2 (2.7%)
Prognosis				
Recovered	232 (68.6%)	57 (89.1%)	154 (77.4%)	21 (28%)
In hospital	74 (21.9%)	7 (10.9%)	45 (22.6%)	22 (29.3%)
Death	32 (9.5%)	0	0	32 (42.7%)

Data are presented as mean ± SD or numbers (%).

COPD, chronic obstructive pulmonary disease; COVID‐19, coronavirus disease 2019; SD, standard deviation.

**Figure 1:**
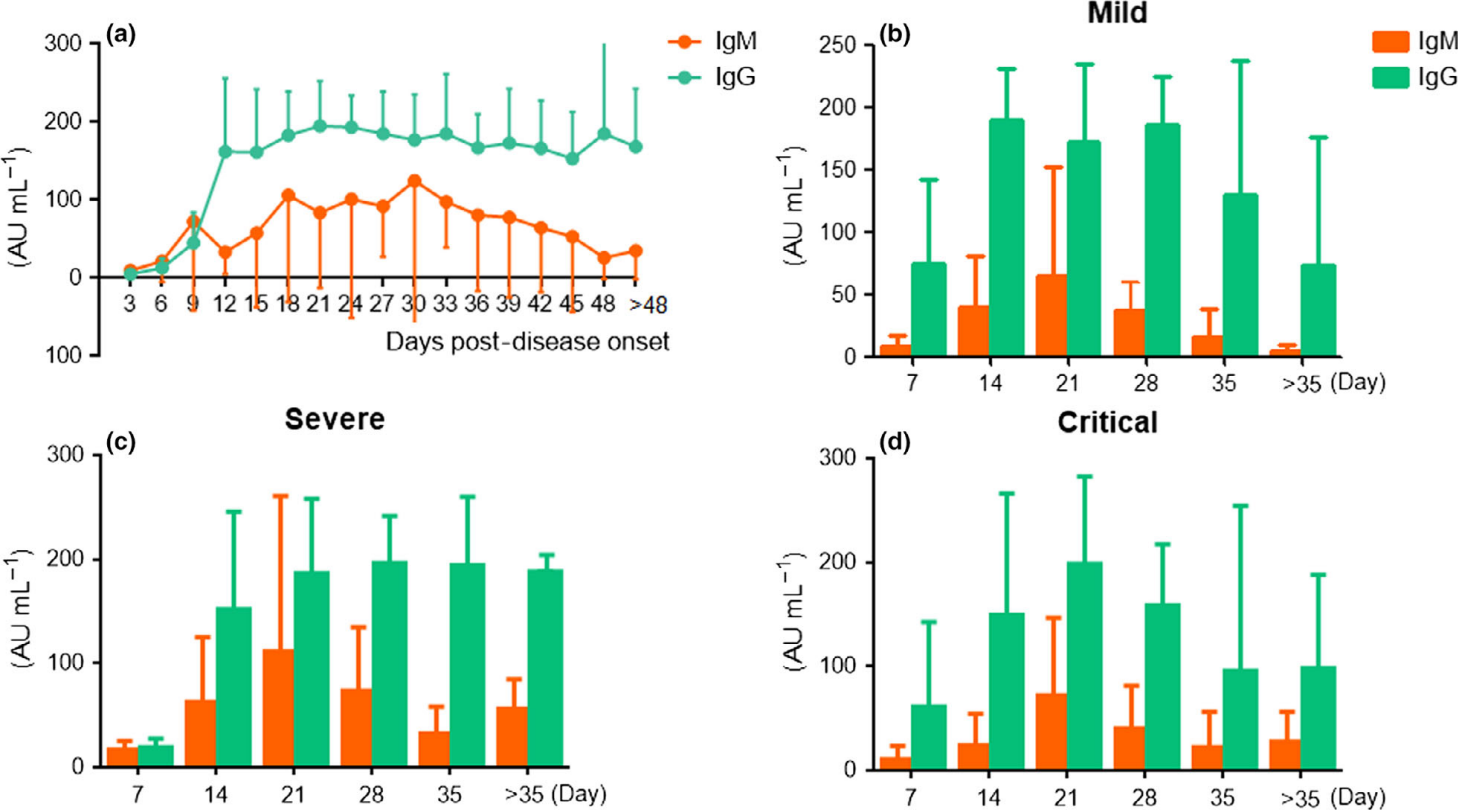
Serological levels of SARS‐CoV‐2‐specific IgM and IgG in COVID‐19 patients. **(a)** The IgM and IgG antibody responses in patients with different disease courses from the symptom onset until the first detection of antibodies are shown. The antibody responses of (**b)** mild, **(c)** severe and **(d)** critical groups across different time‐points were shown. Data are shown as mean ± SD.

We retrospectively analysed the detection results of specific antibody against SARS‐CoV‐2 in COVID‐19 patients. The average levels of IgM and IgG in patients with the same disease courses from symptom onset until the first detection of antibodies are shown in Figure 1a. After SARS‐CoV‐2 infection, the level of IgM increased gradually during the first week, reached its peak after 2 weeks and then reduced to near‐background levels in most patients. Meanwhile, IgG was generated after 1 week, reached its peak level in 3 weeks and was maintained at a high level for an extended period, even over 48 days (Figure 1a). Different patient groups across different time‐points are shown in Figure 1b, c and d. The trends in these subgroups are consistent with those of the data as a whole.

**Figure 2:**
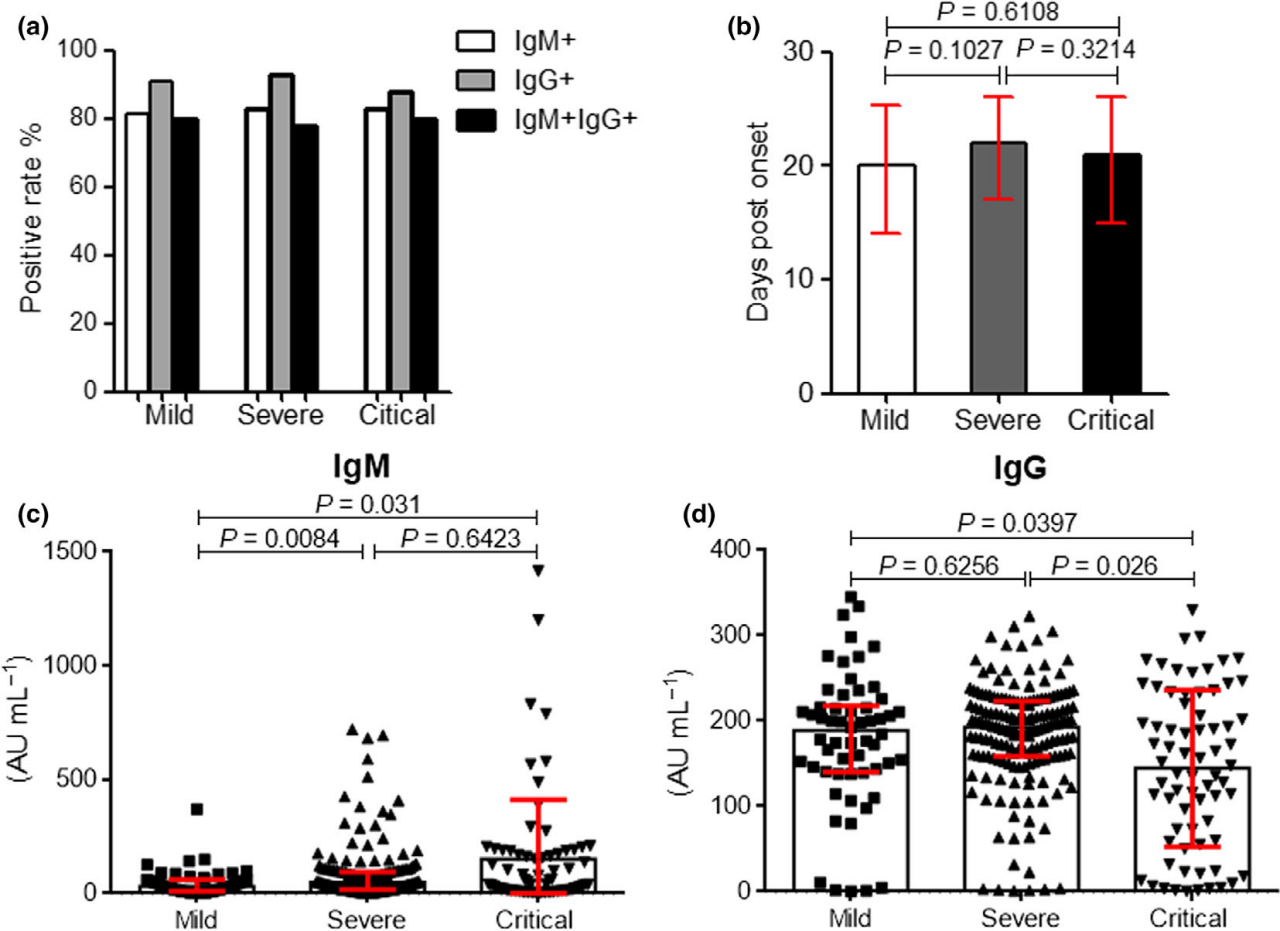
The positive rates and levels of IgM and IgG levels in COVID‐19 patients with different illness severities. **(a)** The rates of patients in whom IgM and/or IgG were detected. (**b)** The median number of days from symptom onset to antibody detection were shown. The median levels of **(c)** IgM and **(d)** IgG in different groups are shown. Results are shown as median and interquartile range and were tested for significance at *P* < 0.05.

In the mild, severe and critical groups, IgM was detected in 81.3%, 82.9% and 82.7% of cases, IgG was detected in 90.6%, 92.7% and 88% of cases, and both IgM and IgG were detected in 79.7%, 77.9% and 80% of cases, respectively (Figure 2a). The median number of days from symptom onset to antibody detection was not significantly different across the mild, severe and critical groups (20.95 ± 9.226 days, 21.9 ± 8.724 days and 20.86 ± 8.126 days, respectively) (Figure 2b). The levels of IgM in the severe and critical groups were higher than those in the mild group (severe vs. mild, *P* = 0.0084; critical vs. mild, *P* = 0.031) (Figure 2c). In contrast, the levels of IgG in the critical group were lower than those in either the mild or severe groups (critical vs. mild, *P* = 0.0397; critical vs. severe, *P* = 0.026) (Figure 2d).

**Figure 3:**
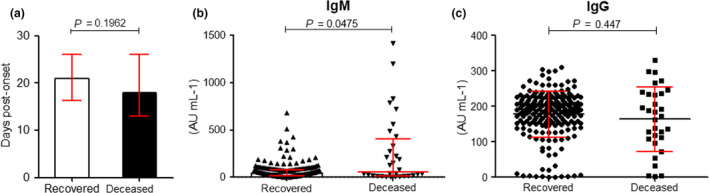
IgM and IgG levels in recovered and deceased patients. **(a)** The median number of days from symptom onset to antibody detection in recovered and deceased groups was shown. The median levels of (**b)** IgM and **(c)** IgG in these groups were shown. Results are shown as median and interquartile range and were tested for significance  at *P* < 0.05.

The antibody levels were further analysed between COVID‐19 cases with different outcomes (recovered or deceased). There was no significant difference in the median numbers of days from symptom onset to antibody detection between the recovered and deceased groups (21.05 ± 7.256 days vs. 19.87 ± 9.383 days, *P* = 0.196) (Figure 3a). Our data show that the IgG levels in these two groups were almost the same (*P* = 0.447), whereas the IgM levels were slightly higher in the deceased group than in the recovered group (*P* = 0.0475) (Figure 3b and c).

**Figure 4:**
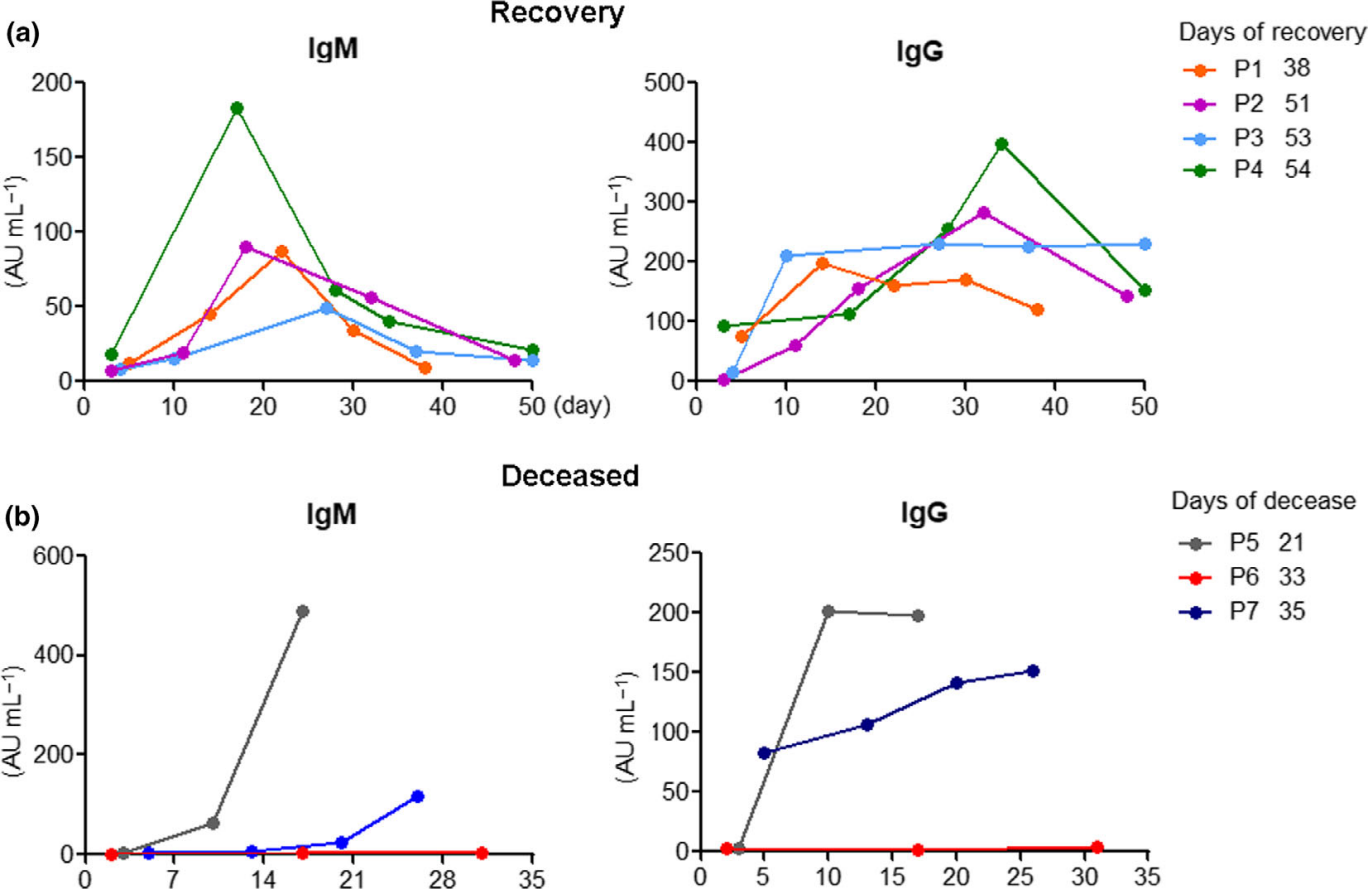
The dynamic change of antibody levels against SARS‐CoV‐2. **(a)** In the three recovered patients (Patients 1–4), longitudinal detection of IgM and IgG levels is shown. (**b)** In the three deceased patients (Patients 5–7), longitudinal detection of IgM and IgG levels is shown.

We also monitored the dynamic changes of IgG and IgM levels in seven patients (one mild case, two severe cases and four critical cases) with different clinical outcomes. The basic characteristics of these patients are shown in Table 2. In the recovered cases (Patients 1–4), the IgM level reached its peak after 2 weeks and then decreased rapidly by 3 weeks. IgG was maintained at a high concentration even after 7 weeks. Of the three deceased cases, IgM and IgG antibodies could be detected in only two of them (Patients 5 and 6). Although IgG was generated in these patients, the IgM level quickly doubled 3–5 days before death. Both IgM and IgG were undetectable in the blood of Patient 7 through the final test on day 31, and the patient died at 35 days after symptom onset (Figure 4). This case was an older patient who might have had generally poor immunity.

**Table 2:** The basic features and clinical outcome of six patients with longitudinal detection of IgM and IgG against SARS‐CoV‐2

Patient	Outcome	Age	Sex	Type group	Signs and symptoms at admission	Comorbidities
P1	Recovered	50	F	Mild	Fever, cough, fatigue	Hypertension
P2	Recovered	50	M	Severe	Fever, chest distress	Cardiovascular disease
P3	Recovered	56	F	Critical	Cough	Hypertension, diabetes
P4	Recovered	68	M	Severe	Fever	Hypertension
P5	Death	55	F	Critical	Fatigue, cough	Cardiovascular disease
P6	Death	70	M	Critical	Fever	Hypertension
P7	Death	84	M	Critical	Fever, cough	Hypertension

## Discussion

In the SARS epidemic, the detection of IgM and IgG allowed for serological diagnosis.^16^ Similar serological responses have been observed in COVID‐19 patients, and the dynamic pattern of these responses is consistent with acute viral infection.^14^ Testing antibodies against SARS‐CoV‐2 is rapid and sensitive for the auxiliary diagnosis of COVID‐19.^17^ Several different detection methods, such as lateral flow immunoassay, CLIA and enzyme‐linked immunosorbent assay, are currently available. The assessment of various reagents in our laboratory revealed high sensitivity and good specificity for CLIA used in the serum diagnosis of COVID‐19. In the present study, the serological responses, that is the levels of both IgM and IgG antibodies, were retrospectively analysed in COVID‐19 patients with different illness severities and outcomes.

During viral infection with SARS‐CoV‐2, the production of specific antibodies against the virus is consistent in most patients, except for immunodeficient patients. IgM can be detected as early as 3 days after infection and provides the first line of humoural immunity defence, after which high‐affinity IgG responses are initiated and play a key role in long‐term immune memory.^22^ SARS‐CoV‐2 is a beta‐coronavirus and shares some similarities with SARS and MERS, because of these similarities, knowledge gained from studies on these other pathogenic coronaviruses can provide insight into the antibody responses that occur during SARS‐CoV‐2 infection.^12^, ^23^ Our data here show that IgM was generated in COVID‐19 patients in 1 week after symptom onset, then reached its peak level in 2–3 weeks, after which the level decreased. Meanwhile, IgG levels increased quickly beginning a little later compared with IgM and were maintained at a high level for 2 months. Therefore, the detectable levels of IgM and IgG antibodies could provide information regarding serological convention over the disease course, as the detection of IgM antibody indicates a recent exposure to SARS‐CoV‐2 and the detection of IgG antibody in the absence of detectable IgM antibody indicates prior virus exposure.

The positive rates of IgM and/or IgG detection were not significantly different among the mild, severe and critical groups. However, quantitative analyses of antibody levels over the disease course revealed that SARS‐CoV‐2‐specific IgM levels were higher and neutralising IgG levels were lower in patients in the critical group, as compared with the other groups, which might be because of high disease activity and/or a compromised immune response in these patients. In contrast, in the mild group patients, IgG was maintained at a high level, while IgM levels gradually decreased when most of the patients were in the recovery state of infection. Furthermore, the level of IgM antibody was higher in the group of deceased cases than that in the group of recovered cases, whereas the IgG level was not significantly different between these groups. The IgM level showed heterogeneity within the group of deceased cases, and some patients had very high IgM levels which might be in the active status of disease or very low IgM levels due to the long disease course . The increased IgM level in the deceased case group might be related to the higher disease severity in these patients and indicate a poor prognosis. Alternately, cytokine storm, severe immune dysfunction and other commobidities might be the important risk factors in these cases.^5^, ^18^, ^21^, ^24^

Notably, the percentage of deceased cases in this study was 9.5%, which is higher than that of previous studies^19^; this is because Tongji Hospital is a designated hospital for admitting severe patients. We also monitored the dynamic change in antibody levels in several recovered and deceased cases. In the four recovered patients (Patients 1–4), IgM levels were low, but IgG was maintained at a high level before discharge, which is consistent with serum conversion as shown in a previous report.^11^ However, the IgM levels in Patients 5 and 6 showed an increased even before the deceased time point. Neither IgG nor IgM was detected in Patient 7 within 31 days from symptom onset, and this patient was deceased on day 35, 4 days after the final antibody test was conducted. Therefore, we speculate that the quantitative results of antibody detection are associated with the severity of COVID‐19 and have potential value for use in predicting the disease prognosis.

Several limitations should be mentioned. First, false‐negative and false‐positive results of antibody detection might affect the analysis among patients with different illness severities and disease courses. The results of this work need to be further validated by studies in a larger number of patients. Second, the time from symptom onset to admission may be long and the data of continuous monitoring in one patient were limited. Third, the relationship between antibody levels and viral copies within the same patients is unknown; this question needs to be studied further.

In conclusion, this study found that anti‐SARS‐CoV‐2 antibody levels differ significantly among COVID‐19 patients with different illness severities and outcomes. Quantitative IgM and IgG assays could play an important role in the diagnosis and prognosis of COVID‐19.

## Methods

### Patients

Between 16 and 25 February 2020, 338 COVID‐19 patients were continuously recruited from Tongji Hospital, Wuhan, China. The confirmed diagnosis of COVID‐19 was defined as a positive result using real‐time RT‐PCR detection from routine nasal and pharyngeal swab specimens. Patient symptoms, signs and laboratory tests during the hospital stay were collected. Fifty‐two healthy controls were also included. This study was approved by the ethical committee of Tongji Hospital, Tongji Medical College, Huazhong University of Science and Technology, Wuhan, China.

### Grouping criteria

The mild cases are those with fever, typical symptoms and pneumonia on chest radiography. Severe cases need to meet one of the following criteria: (1) respiratory distress (respiration rate ≥ 30 times/min); (2) blood oxygen saturation (SpO_2_) ≤ 93% in resting state; and (3) arterial partial pressure of O_2_ to fraction of inspired oxygen (PaO_2_/FiO_2_) ratio ≤ 300 mmHg. Critical cases meet one of the following criteria: (1) respiratory failure requiring mechanical ventilation; (2) shock; and (3) multiple organ dysfunction needing intensive care unit (ICU) treatment. The clinical information related to patient classification was collected from the medical records of the patients.

### Real‐time RT‐PCR

Throat swab or nasal swab specimens from the upper respiratory tract of all patients on admission were collected and maintained in viral transport medium. Sputum specimens were also collected in some patients. SARS‐CoV‐2 infection was confirmed using TaqMan One‐Step RT‐PCR kits from Shanghai Huirui Biotechnology Co., Ltd. (Shanghai, China), and Shanghai BioGerm Medical Biotechnology Co. Ltd. (Shanghai, China), both of which have been approved by the China Food and Drug Administration.

### SARS‐CoV‐2 antibody detection

The IgM and IgG antibodies against SARS‐CoV‐2 in serum specimens were detected using YHLO‐CLIA‐IgG, YHLO‐CLIA‐IgM kits supplied by YHLO (YHLO Biotech Co. Ltd Shenzhen, China), according to the manufacturer's instructions. The recombinant antigens contain nucleoprotein and spike protein of SARS‐CoV‐2. The antibody levels were expressed as arbitrary unit per mL (AU mL^−1^). The results ≥ 10 AU mL^−1^ are reactive (positive), and the results < 10 AU mL^−1^ are nonreactive (negative).

### Statistical analysis

The results are presented as mean ± standard deviation (SD) or median and interquartile range. Differences between groups were analysed using the Mann–Whitney *U*‐test. Statistical analyses were performed using GraphPad Prism version 6 (GraphPad Software Inc., San Diego, CA, USA). Statistical significance was determined to be *P* < 0.05.

## Conflict of interest

The authors declare no conflict of interest.

## Author contributions

**Hongyan Hou:** Data curation; Formal analysis; Project administration; Writing‐original draft. **Ting Wang:** Writing‐original draft. **Bo Zhang:** Data curation; Methodology. **Ying Luo:** Data curation; Software. **Lie Mao:** Formal analysis. **Feng Wang:** Project administration; Writing‐review & editing. **Shiji Wu:** Data curation; Formal analysis. **Ziyong Sun:** Project administration; Writing‐review & editing.
