# Supplementary figures and images for: Detection of IgM and IgG antibodies in patients with coronavirus disease 2019
Source: Clin Transl Immunology. 2020 May 6;9(5):e1136. doi: 10.1002/cti2.1136 (PMC7202656; doi:10.1002/cti2.1136)

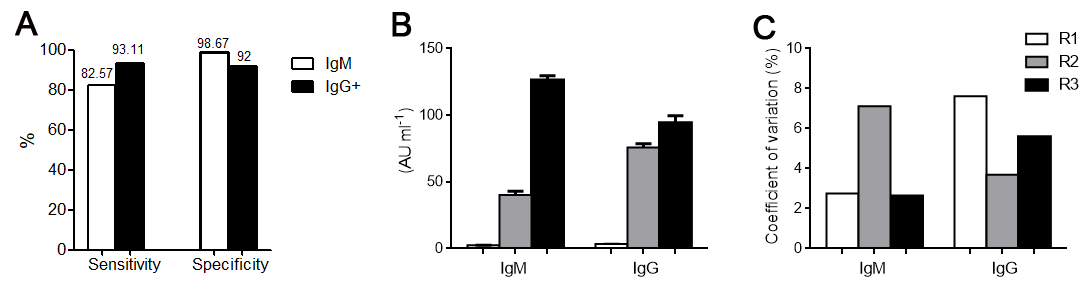

Supplement: Supplementary file 1 [file CTI2-9-e1136-s001.tif]
