# Supplementary material for: Detection of IgM and IgG antibodies in patients with coronavirus disease 2019
Source: Clin Transl Immunology. 2020 May 6;9(5):e1136. doi: 10.1002/cti2.1136 (PMC7202656; doi:10.1002/cti2.1136)
Supplement: Supplementary file 2 [file CTI2-9-e1136-s002.docx]

Supplementary figure 1. The performance of CLIA-YHLO kit of anti-SARS-CoV-2 was assessed in 305 confirmed patients and 142 negative controls in our previous studies. **(a)** The sensitivity and specificity of IgM and IgG was shown. (**b)** Three specimens (Repeated sample 1-3) were repeated ten times for reproducible analysis. The mean ± SD of IgM and IgG levels was shown. **(c)** The CVs of each test are shown in the graph.
